# Supplementary material for: Situating zoonotic diseases in peacebuilding and development theories: Prioritizing zoonoses in Jordan
Source: PLoS One. 2022 Mar 17;17(3):e0265508. doi: 10.1371/journal.pone.0265508 (PMC8929606; doi:10.1371/journal.pone.0265508)
Supplement: S1 Appendix — (DOCX) [file pone.0265508.s001.docx]

**Phase 1 Data**

**Zoonoses in Humans and Animals within Jordan from 2007 - 2016**

*Zoonoses Reported in Animal Populations Within Jordan from 2007 - 2016*

| **Zoonotic Diseases Present in Animals Reported to OIE by Jordan** | | | | | | | | | | |
| --- | --- | --- | --- | --- | --- | --- | --- | --- | --- | --- |
|  | **2007** | **2008** | **2009** | **2010** | **2011** | **2012** | **2013** | **2014** | **2015** | **2016** |
| Acarpisosis of honey bees |  |  |  |  | + | + | + | + | + | + |
| American foulbrood of honey bees |  |  |  |  | + | + | + | + | + | + |
| Anthrax | + | + | + | + | + | + | + | + | + | + |
| avian infectious bronchitis | + | + | + | + | + | + | + | + | + | + |
| Avian mycoplasmosis | + | + | + | + |  |  |  |  |  |  |
| Bluetongue |  |  |  |  |  |  |  |  |  | + |
| Bovine anaplasmosis |  |  |  | + | + | + | + | + | + | + |
| Bovine babesiosis | + | + | + | + | + | + | + | + | + | + |
| BTB | + | + | + | + | + | + | + | + | + | + |
| Bovine viral diarrhea |  |  |  | + |  |  |  |  |  |  |
| Brucellosis | + | + | + | + | + | + | + | + | + | + |
| Camelpox | + |  |  |  |  |  |  |  |  |  |
| Caprine arthritis/encephalitis | + | + | + | + | + | + | + | + | + | + |
| Contagious agalactia | + | + | + | + | + | + | + | + | + | + |
| Crimean Congo hemorrhagic fever |  |  |  |  |  |  |  |  |  | + |
| Echinococcosis | + | + | + | + | + | + | + | + | + | + |
| Chlamydiosis | + | + | + | + | + | + | + | + | + | + |
| Equine piroplasmosis |  | + | + | + | + | + | + | + | + | + |
| EUR foulbrood of honey bees |  |  |  |  | + | + | + | + | + | + |
| Fowl cholera | + | + | + | + |  |  |  |  |  |  |
| IBR/IPIV | + | + | + | + | + | + | + | + | + | + |
| Gumboro | + | + | + | + | + | + | + | + | + | + |
| Leishmaniosis |  |  | + | + | + | + | + | + | + | + |
| Leptospirosis |  |  | + | + | + |  |  |  |  |  |
| Lumpy skin dx |  |  |  |  |  |  | + | + |  | + |
| Maedi-visna | + | + | + | + | + | + | + | + | + | + |
| Mareks disease | + | + | + | + |  |  |  |  |  |  |
| Mycoplasmosis | + | + | + | + |  |  |  |  |  |  |
| Pullorum dx | + | + | + | + |  |  |  |  |  |  |
| Q fever | + | + | + | + |  |  |  | + | + | + |
| Rabies | + | + | + | + | + | + | + | + | + | + |
| Sheep pox | + | + | + | + | + | + | + | + | + | + |
| Surra | + | + | + | + | + | + | + | + | + | + |
| Theileriosis | + | + | + | + |  |  |  |  |  |  |
| Trichomoniasis | + | + | + | + | + |  |  |  |  |  |
| West Nile Fever |  |  |  |  |  | + | + | + | + | + |

*Note.*  Data collected in October 2020 from: World Organization for Animal Health (OIE). (n.d.). World Animal Health Information Database (WAHIS Interface) within the OIE. <https://www.oie.int/wahis_2/public/wahid.php/Country>[information/Zoonoses](https://www.oie.int/wahis_2/public/wahid.php/Countryinformation/Zoonoses)

*Zoonotic Disease Cases in Humans Reported to OIE by Jordan from 2007-2016*

|  | **2007** | **2008** | **2009** | **2010** | **2011** | **2012** | **2013** | **2014** | **2015** | **2016** |
| --- | --- | --- | --- | --- | --- | --- | --- | --- | --- | --- |
| **Monkeypox** | 6816 |  |  |  |  |  |  |  |  |  |
| **Botulism** | 1822 |  |  |  |  |  |  |  |  |  |
| **Brucellosis** | 217 | 111 | 130 | 129 | 119 | 96 | 158 | 273 | 384 | 441 |
| **Leishmaniasis** | 181 | 244 | 148 | 155 | 136 | 103 | 146 | 180 | 257 | 281 |
| **Echinococcosis** | 1 | 3 | 6 |  |  |  |  |  | + |  |
| **Bovine**  **Tuberculosis** | + |  |  |  | 312 | 234 | 365 | 614 | + |  |
| **Toxoplasmosis** | + |  |  | + | + | + | + | + | + |  |
| **Salmonellosis** | + |  |  | + | + | + | + | + | + |  |
| **Q fever** |  |  | 2 |  |  |  |  |  |  |  |
| **Rabies** |  |  |  |  |  | 1 |  | 1 |  | 1 |
| **Anthrax** |  |  |  |  |  |  |  |  | + |  |

*Note.*  Data collected in October 2020 from: World Organization for Animal Health (OIE). (n.d.). World Animal Health Information Database (WAHIS Interface) within the OIE. <https://www.oie.int/wahis_2/public/wahid.php/Country>[information/Zoonoses](https://www.oie.int/wahis_2/public/wahid.php/Countryinformation/Zoonoses)
