# Supplementary material for: Situating zoonotic diseases in peacebuilding and development theories: Prioritizing zoonoses in Jordan
Source: PLoS One. 2022 Mar 17;17(3):e0265508. doi: 10.1371/journal.pone.0265508 (PMC8929606; doi:10.1371/journal.pone.0265508)
Supplement: S2 Appendix — (ZIP) [file pone.0265508.s002.zip › All R Coding .docx]

#Logistic Regression Setup

setwd("~/Desktop")

grouping_data<-read.csv("Data_for_grouping1.csv")

View(grouping_data)

head(grouping_data)

#Numerators

SDAdjusted<-grouping_data[,4]

SDAdjusted

SEAdjusted<-grouping_data[,9]

SEAdjusted

DBAdjusted<-grouping_data[,16]

DBAdjusted

#Denominator

CMAdjusted<-grouping_data[,22]

CMAdjusted

Total<-grouping_data[,2]

Total

SDAdjusted+SEAdjusted+DBAdjusted+CMAdjusted

[1] 1.0000000 1.0000000 1.0000001 1.0000000 1.0000000 0.9999999

num<-c(SDAdjusted,SEAdjusted,DBAdjusted)

num

#Logs

log1<-log(SDAdjusted[1]/(1-(CMAdjusted[1])))

log1

log2<-log(SEAdjusted[1]/(1-(CMAdjusted[1])))

log2

log3<-log(DBAdjusted[1]/(1-CMAdjusted[1]))

log3

log4<-log(SDAdjusted[2]/(1-CMAdjusted[2]))

log4

log5<-log(SEAdjusted[2]/(1-CMAdjusted[2]))

log5

log6<-log(DBAdjusted[2]/(1-CMAdjusted[2]))

log6

log7<-log(SDAdjusted[3]/(1-CMAdjusted[3]))

log7

log8<-log(SEAdjusted[3]/(1-CMAdjusted[3]))

log8

log9<-log(DBAdjusted[3]/(1-CMAdjusted[3]))

log9

log10<-log(SDAdjusted[4]/(1-CMAdjusted[4]))

log10

log11<-log(SEAdjusted[4]/(1-CMAdjusted[4]))

log11

log12<-log(DBAdjusted[4]/(1-CMAdjusted[4]))

log12

log13<-log(SDAdjusted[5]/(1-CMAdjusted[5]))

log13

log14<-log(SEAdjusted[5]/(1-CMAdjusted[5]))

log14

log15<-log(DBAdjusted[5]/(1-CMAdjusted[5]))

log15

log16<-log(SDAdjusted[6]/(1-CMAdjusted[6]))

log16

log17<-log(SEAdjusted[6]/(1-CMAdjusted[6]))

log17

log18<-log(DBAdjusted[6]/(1-CMAdjusted[6]))

log18

response<-c(log1,log2,log3,log4,log5,log6,log7,log8,log9,log10,log11,log12,log13,log14,log15,log16,log17,log18)

response

#Explanatory variables

TBMort<-grouping_data[,5]

MalariaMort<-grouping_data[,6]

HIVMort<-grouping_data[,7]

GDP<-grouping_data[,10]

GINI<-grouping_data[,11]

GINIcoeff<-grouping_data[,12]

MedianHI<-c(1530,2075,2346,1870,1983,2154)

Meatcons<-grouping_data[,14]

Vet<-grouping_data[,17]

Hospitalbeds<-grouping_data[,18]

GovHealthExp<-grouping_data[,19]

CHE<-grouping_data[,20]

MalariaGovExp<-grouping_data[,23]

OIEBTB<-grouping_data[,24]

#Intercepts Example Vector

int1 is the intercept of SD of 6 countries (int2 is SE, int3 is DB)

int1<-c(1,0,0,1,0,0,1,0,0,1,0,0,1,0,0,1,0,0)

int2<-c(0,1,0,0,1,0,0,1,0,0,1,0,0,1,0,0,1,0)

int3<-c(0,0,1,0,0,1,0,0,1,0,0,1,0,0,1,0,0,1)

#Example Vector Variables

TBM<-c(TBMort[1],TBMort[1],TBMort[1],TBMort[2],TBMort[2],TBMort[2],TBMort[3],TBMort[3],TBMort[3],TBMort[4],TBMort[4],TBMort[4],TBMort[5],TBMort[5],TBMort[5],TBMort[6],TBMort[6],TBMort[6])

MM<-c(MalariaMort[1],MalariaMort[1],MalariaMort[1], MalariaMort[2], MalariaMort[2], MalariaMort[2], MalariaMort[3], MalariaMort[3], MalariaMort[3], MalariaMort[4], MalariaMort[4], MalariaMort[4], MalariaMort[5], MalariaMort[5], MalariaMort[5], MalariaMort[6], MalariaMort[6], MalariaMort[6])

HIVM<-c(HIVMort[1], HIVMort[1], HIVMort[1], HIVMort[2], HIVMort[2], HIVMort[2], HIVMort[3], HIVMort[3], HIVMort[3], HIVMort[4], HIVMort[4], HIVMort[4], HIVMort[5], HIVMort[5], HIVMort[5], HIVMort[6], HIVMort[6], HIVMort[6])

GDP<-c(GDP[1], GDP[1], GDP[1],GDP[2],GDP[2],GDP[2],GDP[3],GDP[3],GDP[3],GDP[4],GDP[4],GDP[4],GDP[5],GDP[5],GDP[5],GDP[6],GDP[6],GDP[6])

Gini<-c(GINI[1],GINI[1],GINI[1],GINI[2],GINI[2],GINI[2],GINI[3],GINI[3],GINI[3],GINI[4],GINI[4],GINI[4],GINI[5],GINI[5],GINI[5],GINI[6],GINI[6],GINI[6])

GINIc<-c(GINIcoeff[1], GINIcoeff[1], GINIcoeff[1], GINIcoeff[2], GINIcoeff[2], GINIcoeff[2],GINIcoeff[3],GINIcoeff[3],GINIcoeff[3],GINIcoeff[4],GINIcoeff[4],GINIcoeff[4],GINIcoeff[5],GINIcoeff[5],GINIcoeff[5],GINIcoeff[6],GINIcoeff[6],GINIcoeff[6])

MHI<-c(MedianHI[1], MedianHI[1], MedianHI[1], MedianHI[2], MedianHI[2], MedianHI[2],MedianHI[3], MedianHI[3], MedianHI[3], MedianHI[4], MedianHI[4], MedianHI[4], MedianHI[5], MedianHI[5], MedianHI[5], MedianHI[6], MedianHI[6], MedianHI[6])

Vets<-c(Vet[1], Vet[1], Vet[1], Vet[2], Vet[2], Vet[2], Vet[3], Vet[3], Vet[3], Vet[4], Vet[4], Vet[4], Vet[5], Vet[5], Vet[5], Vet[6], Vet[6], Vet[6])

Meat<-c(Meatcons[1],Meatcons[1],Meatcons[1],Meatcons[2],Meatcons[2],Meatcons[2],Meatcons[3],Meatcons[3],Meatcons[3],Meatcons[4],Meatcons[4],Meatcons[4],Meatcons[5],Meatcons[5],Meatcons[5],Meatcons[6],Meatcons[6],Meatcons[6])

Hosp<-c(Hospitalbeds[1],Hospitalbeds[1],Hospitalbeds[1],Hospitalbeds[2],Hospitalbeds[2],Hospitalbeds[2],Hospitalbeds[3],Hospitalbeds[3],Hospitalbeds[3],Hospitalbeds[4],Hospitalbeds[4],Hospitalbeds[4],Hospitalbeds[5],Hospitalbeds[5],Hospitalbeds[5],Hospitalbeds[6],Hospitalbeds[6],Hospitalbeds[6])

GHE<-c(GovHealthExp[1],GovHealthExp[1],GovHealthExp[1],GovHealthExp[2],GovHealthExp[2],GovHealthExp[2],GovHealthExp[3],GovHealthExp[3],GovHealthExp[3],GovHealthExp[4],GovHealthExp[4],GovHealthExp[4],GovHealthExp[5],GovHealthExp[5],GovHealthExp[5],GovHealthExp[6],GovHealthExp[6],GovHealthExp[6])

CHE<-c(CHE[1],CHE[1],CHE[1],CHE[2],CHE[2],CHE[2],CHE[3],CHE[3],CHE[3],CHE[4],CHE[4],CHE[4],CHE[5],CHE[5],CHE[5],CHE[6],CHE[6],CHE[6])

MGE<-c(MalariaGovExp[1],MalariaGovExp[1],MalariaGovExp[1],MalariaGovExp[2],MalariaGovExp[2],MalariaGovExp[2],MalariaGovExp[3],MalariaGovExp[3],MalariaGovExp[3],MalariaGovExp[4],MalariaGovExp[4],MalariaGovExp[4],MalariaGovExp[5],MalariaGovExp[5],MalariaGovExp[5],MalariaGovExp[6],MalariaGovExp[6],MalariaGovExp[6])

OIE<-c(OIEBTB[1],OIEBTB[1],OIEBTB[1],OIEBTB[2],OIEBTB[2],OIEBTB[2],OIEBTB[3],OIEBTB[3],OIEBTB[3],OIEBTB[4],OIEBTB[4],OIEBTB[4],OIEBTB[5],OIEBTB[5],OIEBTB[5],OIEBTB[6],OIEBTB[6],OIEBTB[6])

#Baseline

lm.baseline<-lm(response~0+int1+int2+int3)

summary(lm.baseline)

#create residual vs. fitted plot

plot(fitted(lm.baseline), resid(lm.baseline), xlab='Fitted Values', ylab='Residuals')

#add a horizontal line at 0

abline(0,0)

fitted(lm.baseline)

#We don’t know if they’re distributed with equal variance throughout the plot.

#To formally test for heteroscedasticity, we can perform a Breusch-Pagan test:

install.packages("lmtest")

library(lmtest)

bptest(lm.baseline)

#studentized Breusch-Pagan test

data: lm.baseline

BP = 5.0177, df = 2, p-value = 0.08136

Since the p-value from the test is 0.08136 we will not reject the null hypothesis and conclude that heteroscedasticity is a not problem in this model.

#Just in case, I perform weighted least squares by defining the weights in such a way that the observations with lower variance are given more weight:

wt <- 1 / lm(abs(lm.baseline$residuals) ~ lm.baseline$fitted.values)$fitted.values^2

lm.base.weighted<-lm(response~0+int1+int2+int3,weights = wt)

summary(lm.base.weighted)

#No significant change.

#Regression analysis with all variables:

MM, MHI, Vets

TBM, HIVM

GDP, Gini, OIE, Meat

Hosp, GHE, CHE

lm1<-lm(response~0+int1+int2+int3+MM+MHI+Vets)

summary(lm1)

lm2<-lm(response~0+int1+int2+int3+MM+MHI+TBM)

summary(lm2)

lm3<-lm(response~0+int1+int2+int3+MM+MHI+HIVM)

summary(lm3)

lm4<-lm(response~0+int1+int2+int3+MM+MHI+GDP)

summary(lm4)

lm5<-lm(response~0+int1+int2+int3+MM+MHI+Gini)

summary(lm5)

lm6<-lm(response~0+int1+int2+int3+MM+MHI+OIE)

summary(lm6)

lm7<-lm(response~0+int1+int2+int3+MM+MHI+Meat)

summary(lm7)

lm8<-lm(response~0+int1+int2+int3+MM+MHI+Hosp)

summary(lm8)

lm9<-lm(response~0+int1+int2+int3+MM+MHI+GHE)

summary(lm9)

lm10<-lm(response~0+int1+int2+int3+MM+MHI+CHE)

summary(lm10)

lm11<-lm(response~0+int1+int2+int3+MM+Vets+TBM)

summary(lm11)

lm12<-lm(response~0+int1+int2+int3+MM+Vets+HIVM)

summary(lm12)

lm13<-lm(response~0+int1+int2+int3+MM+Vets+GDP)

summary(lm13)

lm14<-lm(response~0+int1+int2+int3+MM+Vets+Gini)

summary(lm14)

lm15<-lm(response~0+int1+int2+int3+MM+Vets+OIE)

summary(lm15)

lm16<-lm(response~0+int1+int2+int3+MM+Vets+Hosp)

summary(lm16)

lm17<-lm(response~0+int1+int2+int3+MM+Vets+GHE)

summary(lm17)

lm18<-lm(response~0+int1+int2+int3+MM+Vets+CHE)

summary(lm18)

lm19<-lm(response~0+int1+int2+int3+MM+TBM+HIVM)

summary(lm19)

lm20<-lm(response~0+int1+int2+int3+MM+TBM+GDP)

summary(lm20)

lm21<-lm(response~0+int1+int2+int3+MM+TBM+Gini)

summary(lm21)

lm1<-lm(response~0+int1+int2+int3+MM+TBM+OIE)

summary(lm1)

lm1<-lm(response~0+int1+int2+int3+MM+TBM+Meat, data=expvallexc14.df)

summary(lm1)

#Only 2 barely sig

lm22<-lm(response~0+int1+int2+int3+MM+TBM+Hosp, data=expvallexc14.df)

summary(lm22)

lm23<-lm(response~0+int1+int2+int3+MM+TBM+GHE, data=expvallexc14.df)

summary(lm23)

#Only 1 and 2

lm24<-lm(response~0+int1+int2+int3+MM+TBM+CHE, data=expvallexc14.df)

summary(lm24)

lm25<-lm(response~0+int1+int2+int3+MM+HIVM+GDP, data=expvallexc14.df)

summary(lm25)

lm26<-lm(response~0+int1+int2+int3+MM+HIVM+Gini, data=expvallexc14.df)

summary(lm26)

#Only 2 sig barely

lm27<-lm(response~0+int1+int2+int3+MM+HIVM+OIE, data=expvallexc14.df)

summary(lm27)

#Only 1 and 2

lm28<-lm(response~0+int1+int2+int3+MM+HIVM+Meat, data=expvallexc14.df)

summary(lm28)

lm29<-lm(response~0+int1+int2+int3+MM+HIVM+Hosp, data=expvallexc14.df)

summary(lm29)

lm30<-lm(response~0+int1+int2+int3+MM+HIVM+GHE, data=expvallexc14.df)

summary(lm30)

lm31<-lm(response~0+int1+int2+int3+MM+HIVM+CHE, data=expvallexc14.df)

summary(lm31)

lm32<-lm(response~0+int1+int2+int3+MM+GDP+Gini, data=expvallexc14.df)

summary(lm32)

#Only 1 barely and 2

lm33<-lm(response~0+int1+int2+int3+MM+GDP+OIE, data=expvallexc14.df)

summary(lm33)

#Only 1 barely and2

lm34<-lm(response~0+int1+int2+int3+MM+GDP+Meat, data=expvallexc14.df)

summary(lm34)

lm35<-lm(response~0+int1+int2+int3+MM+GDP+Hosp, data=expvallexc14.df)

summary(lm35)

lm36<-lm(response~0+int1+int2+int3+MM+GDP+GHE, data=expvallexc14.df)

summary(lm36)

lm37<-lm(response~0+int1+int2+int3+MM+GDP+CHE, data=expvallexc14.df)

summary(lm37)

lm38<-lm(response~0+int1+int2+int3+MM+Gini+OIE, data=expvallexc14.df)

summary(lm38)

#None sig only 3 barely

lm39<-lm(response~0+int1+int2+int3+MM+Gini+Meat, data=expvallexc14.df)

summary(lm39)

#None sig

lm40<-lm(response~0+int1+int2+int3+MM+Gini+Hosp, data=expvallexc14.df)

summary(lm40)

#None sig

lm41<-lm(response~0+int1+int2+int3+MM+Gini+GHE, data=expvallexc14.df)

summary(lm41)

#None sig

lm42<-lm(response~0+int1+int2+int3+MM+Gini+CHE, data=expvallexc14.df)

summary(lm42)

#Only 2 sig

lm43<-lm(response~0+int1+int2+int3+MM+OIE+Meat, data=expvallexc14.df)

summary(lm43)

lm44<-lm(response~0+int1+int2+int3+MM+OIE+Hosp, data=expvallexc14.df)

summary(lm44)

lm45<-lm(response~0+int1+int2+int3+MM+OIE+GHE, data=expvallexc14.df)

summary(lm45)

lm46<-lm(response~0+int1+int2+int3+MM+OIE+CHE, data=expvallexc14.df)

summary(lm46)

lm47<-lm(response~0+int1+int2+int3+MM+Meat+Hosp, data=expvallexc14.df)

summary(lm47)

lm48<-lm(response~0+int1+int2+int3+MM+Meat+GHE, data=expvallexc14.df)

summary(lm48)

lm49<-lm(response~0+int1+int2+int3+MM+Meat+CHE, data=expvallexc14.df)

summary(lm49)

lm50<-lm(response~0+int1+int2+int3+MM+Hosp+GHE)

summary(lm50)

##LM 51-100

lm51<-lm(response~0+int1+int2+int3+MM+Hosp+CHE, data=expvallexc14.df)

summary(lm51)

lm52<-lm(response~0+int1+int2+int3+MM+GHE+CHE, data=expvallexc14.df)

summary(lm52)

lm3<-lm(response~0+int1+int2+int3+MHI+Vets+TBM, data=expvallexc14.df)

summary(lm3)

lm4<-lm(response~0+int1+int2+int3+MHI+Vets+HIVM, data=expvallexc14.df)

summary(lm4)

lm5<-lm(response~0+int1+int2+int3+MHI+Vets+GDP, data=expvallexc14.df)

summary(lm5)

lm6<-lm(response~0+int1+int2+int3+MHI+Vets+Gini, data=expvallexc14.df)

summary(lm6)

#Only int2 sig

lm7<-lm(response~0+int1+int2+int3+MHI+Vets+OIE, data=expvallexc14.df)

summary(lm7)

lm8<-lm(response~0+int1+int2+int3+MHI+Vets+CHE, data=expvallexc14.df)

summary(lm8)

lm9<-lm(response~0+int1+int2+int3+MHI+TBM+HIM, data=expvallexc14.df)

summary(lm9)

#None sig

lm10<-lm(response~0+int1+int2+int3+MHI+TBM+GDP, data=expvallexc14.df)

summary(lm10)

lm11<-lm(response~0+int1+int2+int3+MHI+TBM+Gini, data=expvallexc14.df)

summary(lm11)

#2 barely sig

lm12<-lm(response~0+int1+int2+int3+MHI+TBM+OIE, data=expvallexc14.df)

summary(lm12)

#2 barely sig

lm13<-lm(response~0+int1+int2+int3+MHI+TBM+Meat, data=expvallexc14.df)

summary(lm13)

lm14<-lm(response~0+int1+int2+int3+MHI+TBM+Hosp, data=expvallexc14.df)

summary(lm14)

lm15<-lm(response~0+int1+int2+int3+MHI+TBM+GHE, data=expvallexc14.df)

summary(lm15)

lm16<-lm(response~0+int1+int2+int3+MHI+TBM+CHE, data=expvallexc14.df)

summary(lm16)

lm17<-lm(response~0+int1+int2+int3+MHI+HIVM+GDP, data=expvallexc14.df)

summary(lm17)

lm18<-lm(response~0+int1+int2+int3+MHI+HIVM+Gini, data=expvallexc14.df)

summary(lm18)

# Only 2 barely sig

lm19<-lm(response~0+int1+int2+int3+MHI+HIVM+OIE, data=expvallexc14.df)

summary(lm19)

lm20<-lm(response~0+int1+int2+int3+MHI+HIVM+Meat, data=expvallexc14.df)

summary(lm20)

lm21<-lm(response~0+int1+int2+int3+MHI+HIVM+Hosp, data=expvallexc14.df)

summary(lm21)

lm1<-lm(response~0+int1+int2+int3+MHI+HIVM+GHE, data=expvallexc14.df)

summary(lm1)

lm1<-lm(response~0+int1+int2+int3+MHI+HIVM+CHE, data=expvallexc14.df)

summary(lm1)

lm22<-lm(response~0+int1+int2+int3+MHI+GDP+Gini, data=expvallexc14.df)

summary(lm22)

lm23<-lm(response~0+int1+int2+int3+MHI+GDP+OIE, data=expvallexc14.df)

summary(lm23)

lm24<-lm(response~0+int1+int2+int3+MHI+GDP+Meat, data=expvallexc14.df)

summary(lm24)

#Only 1 and 2

lm25<-lm(response~0+int1+int2+int3+MHI+GDP+Hosp, data=expvallexc14.df)

summary(lm25)

lm26<-lm(response~0+int1+int2+int3+MHI+GDP+GHE, data=expvallexc14.df)

summary(lm26)

lm27<-lm(response~0+int1+int2+int3+MHI+GDP+CHE, data=expvallexc14.df)

summary(lm27)

lm28<-lm(response~0+int1+int2+int3+MHI+Gini+OIE, data=expvallexc14.df)

summary(lm28)

#Only 2 and1 barely

lm29<-lm(response~0+int1+int2+int3+MHI+Gini+Meat, data=expvallexc14.df)

summary(lm29)

#None sig

lm30<-lm(response~0+int1+int2+int3+MHI+Gini+Hosp, data=expvallexc14.df)

summary(lm30)

#None sig

lm31<-lm(response~0+int1+int2+int3+MHI+Gini+GHE, data=expvallexc14.df)

summary(lm31)

#Only 2 and 1 barely

lm32<-lm(response~0+int1+int2+int3+MHI+Gini+CHE, data=expvallexc14.df)

summary(lm32)

lm33<-lm(response~0+int1+int2+int3+MHI+OIE+Meat, data=expvallexc14.df)

summary(lm33)

lm34<-lm(response~0+int1+int2+int3+MHI+OIE+Hosp, data=expvallexc14.df)

summary(lm34)

lm35<-lm(response~0+int1+int2+int3+MHI+OIE+GHE, data=expvallexc14.df)

summary(lm35)

lm36<-lm(response~0+int1+int2+int3+MHI+OIE+CHE, data=expvallexc14.df)

summary(lm36)

lm37<-lm(response~0+int1+int2+int3+MHI+OIE+CHE, data=expvallexc14.df)

summary(lm37)

lm38<-lm(response~0+int1+int2+int3+MHI+Meat+Hosp, data=expvallexc14.df)

summary(lm38)

lm39<-lm(response~0+int1+int2+int3+MHI+Meat+GHE, data=expvallexc14.df)

summary(lm39)

#Only 2

lm40<-lm(response~0+int1+int2+int3+MHI+Meat+CHE, data=expvallexc14.df)

summary(lm40)

#Only 2

lm41<-lm(response~0+int1+int2+int3+MHI+Hosp + GHE, data=expvallexc14.df)

summary(lm41)

lm42<-lm(response~0+int1+int2+int3+MHI+Hosp+CHE, data=expvallexc14.df)

summary(lm42)

lm43<-lm(response~0+int1+int2+int3+MHI+GHE+CHE, data=expvallexc14.df)

summary(lm43)

lm44<-lm(response~0+int1+int2+int3+Vets+TBM+HIVM, data=expvallexc14.df)

summary(lm44)

lm45<-lm(response~0+int1+int2+int3+Vets +TBM+GDP, data=expvallexc14.df)

summary(lm45)

lm46<-lm(response~0+int1+int2+int3+Vets+TBM+Gini data=expvallexc14.df)

summary(lm46)

lm47<-lm(response~0+int1+int2+int3+Vets+TBM+OIE, data=expvallexc14.df)

summary(lm47)

lm48<-lm(response~0+int1+int2+int3+Vets+TBM+Meat, data=expvallexc14.df)

summary(lm48)

#Only 1 and 2

lm49<-lm(response~0+int1+int2+int3+Vets+TBM+Hosp, data=expvallexc14.df)

summary(lm49)

lm50<-lm(response~0+int1+int2+int3+Vets+TBM+GHE, data=expvallexc14.df)

summary(lm50)

LM 101-150

MM, MHI, Vets

TBM, HIVM

GDP, Gini, OIE, Meat

Hosp, GHE, CHE

lm101<-lm(response~0+int1+int2+int3+Vets+TBM+CHE, data=expvallexc14.df)

summary(lm101)

lm52<-lm(response~0+int1+int2+int3+Vets+HIVM+GDP, data=expvallexc14.df)

summary(lm52)

lm3<-lm(response~0+int1+int2+int3+Vets+HIVM+Gini, data=expvallexc14.df)

summary(lm3)

#None sig

lm4<-lm(response~0+int1+int2+int3+Vets+HIVM+OIE, data=expvallexc14.df)

summary(lm4)

lm5<-lm(response~0+int1+int2+int3+Vets+HIVM+Meat, data=expvallexc14.df)

summary(lm5)

lm6<-lm(response~0+int1+int2+int3+Vets+HIVM+Hosp, data=expvallexc14.df)

summary(lm6)

lm7<-lm(response~0+int1+int2+int3+Vets+HIVM+GHE, data=expvallexc14.df)

summary(lm7)

lm8<-lm(response~0+int1+int2+int3+Vets+HIVM+CHE, data=expvallexc14.df)

summary(lm8)

lm9<-lm(response~0+int1+int2+int3+Vets+GDP+Gini, data=expvallexc14.df)

summary(lm9)

#Only 2 sig 1 barely

lm10<-lm(response~0+int1+int2+int3+Vets+GDP+OIE, data=expvallexc14.df)

summary(lm10)

lm11<-lm(response~0+int1+int2+int3+Vets+GDP+CHE, data=expvallexc14.df)

summary(lm11)

#2 barely sig

lm12<-lm(response~0+int1+int2+int3+Vets+Gini+OIE, data=expvallexc14.df)

summary(lm12)

#Only 2

lm13<-lm(response~0+int1+int2+int3+Vets+Gini +Meat, data=expvallexc14.df)

summary(lm13)

#None sig

lm14<-lm(response~0+int1+int2+int3+Vets+Gini+Hosp, data=expvallexc14.df)

summary(lm14)

#none

lm15<-lm(response~0+int1+int2+int3+Vets+Gini+GHE, data=expvallexc14.df)

summary(lm15)

#none

lm16<-lm(response~0+int1+int2+int3+Vets+Gini+CHE, data=expvallexc14.df)

summary(lm16)

#Only 2

lm17<-lm(response~0+int1+int2+int3+Vets+OIE+Meat, data=expvallexc14.df)

summary(lm17)

lm18<-lm(response~0+int1+int2+int3+Vets+OIE+Hosp, data=expvallexc14.df)

summary(lm18)

lm19<-lm(response~0+int1+int2+int3+Vets+OIE+GHE, data=expvallexc14.df)

summary(lm19)

lm20<-lm(response~0+int1+int2+int3+Vets+OIE+CHE, data=expvallexc14.df)

summary(lm20)

lm21<-lm(response~0+int1+int2+int3+MHI+Vets+Meat+Hosp, data=expvallexc14.df)

summary(lm21)

#Only 2

lm1<-lm(response~0+int1+int2+int3+Vets+Meat+GHE, data=expvallexc14.df)

summary(lm1)

lm1<-lm(response~0+int1+int2+int3+Vets+Meat+CHE, data=expvallexc14.df)

summary(lm1)

lm22<-lm(response~0+int1+int2+int3+Vets+Hosp+GHE)

summary(lm22)

lm23<-lm(response~0+int1+int2+int3+Vets+Hosp+CHE, data=expvallexc14.df)

summary(lm23)

lm24<-lm(response~0+int1+int2+int3+Vets+GHE+CHE, data=expvallexc14.df)

summary(lm24)

lm25<-lm(response~0+int1+int2+int3+TBM+HIVM+GDP)

summary(lm25)

lm26<-lm(response~0+int1+int2+int3+TBM+HIVM+Gini)

summary(lm26)

CFA

library(corpcor)

library(dplyr)

library(lavaan)

library(psych)

library(semPlot)

library(nFactors)

library(MVN)

library(metaSEM)

library(knitr)

setwd("~/Desktop")

data.all<-read.csv("variables4copycopy.csv", stringsAsFactors = FALSE)

datadf<-data.frame(data.all)

##Dataprep

df1<-lapply(datadf, as.numeric)

View(df1)

print(df1)

str(df1)

df.jm<-data.frame(df1)

df.jms<-scale(df.jm)

df.jms1<-data.frame(df.jms)

df.jms1

summary(svd(df.jm))

df.jmsSD<-data.frame(df.jms[, c(3:11)])

df.jmsSE<-data.frame(df.jms[, c(11:22)])

df.jmsDB<-data.frame(df.jms[, c(22:33)])

df.jmsCM<-data.frame(df.jms[, c(35:46)])

df.jmALLFIT<-data.frame(df.jms[, c(7,9,10,12,17,19,22,24,25,27,35,43,45)])

df.jmALLFIT

## Correlation checks

jmcor<-cor(df.jm)

cor.plot(jmcor)

round(cor(df.jm, use="complete.obs"),2)

lowerCor(df.jms, use="pairwise.complete.obs")

## Factor prep

ap <- parallel(subject=nrow(df.jms),var=ncol(df.jms),

rep=100,cent=.05)

nS <- nScree(x=ev$values, aparallel=ap$eigen$qevpea)

plotnScree(nS)

## Best number of factors would be around 5

## EFA

EFAjm<-fa(df.jms, nfactors=5)

summary(EFAjm)

print(EFAjm)

print(EFAjm$loadings,cutoff = 0.3)

print(efjm1)

fa.diagram(EFAjm)

##test univariate normality

result<-mvn(data=df.jmsSE, mvnTest="royston", univariatePlot="qqplot")

result$univariateNormality

result1<-mvn(data=df.jmsSE,mvnTest="royston",univariateTest="SW",desc=TRUE)

result1$multivariateNormality

result<-mvn(data=df.jmsSD, mvnTest="royston", univariatePlot="qqplot")

result$univariateNormality

result1<-mvn(data=df.jmsSD,mvnTest="royston",univariateTest="SW",desc=TRUE)

result1$multivariateNormality

result<-mvn(data=df.jmsDB, mvnTest="royston", univariatePlot="qqplot")

result$univariateNormality

result1<-mvn(data=df.jmsDB,mvnTest="royston",univariateTest="SW",desc=TRUE)

result1$multivariateNormality

result<-mvn(data=df.jmsCM, mvnTest="royston", univariatePlot="qqplot")

result$univariateNormality

result1<-mvn(data=df.jmsCM,mvnTest="royston",univariateTest="SW",desc=TRUE)

result1$multivariateNormality

##GDPMilit and Milkcons NO for univ normality

### CFA

## SD

SD.model<-'SD=~MInc + InfantM + DalysCD + Hosp'

SDfit<-cfa(SD.model, data=df.jms1, check.gradient=FALSE, std.lv=TRUE)

summary(SDfit, fit.measures=TRUE)

parameterestimates(SDfit, standardized=TRUE)

semPaths(SDfit, "std")

### SE

setwd("~/Desktop")

data.all2<-read.csv("variables4copycopy.csv", stringsAsFactors = FALSE)

datadf2<-data.frame(data.all2)

View(datadf2)

##Creation and scaling of dataframe

df2<-lapply(datadf2, as.numeric)

View(df1)

print(df1)

df.jm2<-data.frame(df2)

df.jm

df.jms2<-scale(df.jm2)

df.jms12<-data.frame(df.jms2)

df.jms12

resultDI<-mvn(data=df.jms12[20:30], mvnTest="royston", univariatePlot="qqplot")

resultDI$univariateNormality

result1<-mvn(data=df.jms12[45:52],mvnTest="royston",univariateTest="SW",desc=TRUE)

result1$multivariateNormality

SE.mod<-'SE=~ Ruralpop + Sugar + Dem + GDPAgr'

SEfit112<-cfa(SE.mod, data=df.jms12, check.gradient=FALSE, std.lv=TRUE)

summary(SEfit112, fit.measures=TRUE)

semPaths(SEfit, "std")

###ENDSE

##DB

DB.model<-'DB=~Cattlepercap + BovineMeatCons + Milkcons + UnsafeSan'

DBfit<-cfa(DB.model, data=df.jms1, check.gradient=FALSE, std.lv=TRUE)

summary(DBfit, fit.measures=TRUE)

parameterestimates(DBfit, standardized=TRUE)

semPaths(DBfit, "std")

##CM

CM.model<-'CM=~OIE+Nets+Tbm+Vet'

CMfit<-cfa(CM.model, data=df.jms1, check.gradient=FALSE, std.lv=TRUE)

summary(CMfit, fit.measures=TRUE)

parameterestimates(CMfit, standardized=TRUE)

semPaths(CMfit, "std")

OTHER

##Normalizing Raw Scores

tooldata<-c(2.33, 2.28, 2.27, 2.07,2.07,2.07,1.82,1.79,1.78,1.71,1.18)

td<-data.frame(tooldata)

normalized<-function(y) {

x<-y[!is.na(y)]

x<-(x - min(x)) / (max(x) - min(x))

y[!is.na(y)]<-x

return(y)

}

apply(td,2,normalized)

## Euclidian Distances

setwd("~/Desktop")

CANDSD<-read.csv("CandSD.csv", stringsAsFactors = FALSE)

CANDSDdf<-data.frame(CANDSD)

View(CANDSDdf)

dist.euclSD <- dist(CANDSDdf[c(5:5)], method = "euclidean")

summary(dist.euclSD)

dist.euclSD

setwd("~/Desktop")

CANDSE<-read.csv("CandSE.csv", stringsAsFactors = FALSE)

CANDSEdf<-data.frame(CANDSE)

View(CANDSEdf)

dist.euclSE <- dist(CANDSEdf[c(5:5)], method = "euclidean")

summary(dist.euclSD)

dist.euclSE

setwd("~/Desktop")

CANDDB<-read.csv("CandDB.csv", stringsAsFactors = FALSE)

CANDDBdf<-data.frame(CANDDB)

View(CANDDB)

dist.euclDB <- dist(CANDDBdf[c(6:6)], method = "euclidean")

summary(dist.euclDB)

dist.euclDB

CANDDBdf2<-(data.frame(CANDDBdf[-c(3,4,6,9),]))

View(CANDDBdf2)

dist.euclDB2<-dist(CANDDBdf2[c(3:3)], method = "euclidean")

dist.euclDB2

CANDCM<-read.csv("CandCM.csv", stringsAsFactors = FALSE)

CANDCMdf<-data.frame(CANDCM)

View(CANDCM)

dist.euclCM <- dist(CANDCMdf[c(4:4)], method = "euclidean")

summary(dist.euclCM)

dist.euclCM

##Eucl Hofstede

setwd("~/Desktop")

hofstede<-read.csv("Untitled spreadsheet - Sheet1.csv", stringsAsFactors = FALSE)

hofstededf<-data.frame(hofstede)

View(hofstede)

hofsteddfnous<-hofstededf[-c(7),]

hofsteddfnous

dist.eucl <- dist(hofstededf[c(2:5)], method = "euclidean")

dist.eucl

dist.eucl1<-dist(hofsteddfnous[c(2:5)])

str(hofstededf)

dist.cor<-get_dist(hofstededf[c(2:5)], method = "pearson")

dist.cor

hofsteddfnous[c(2:5)]

# Display a subset

round(as.matrix(dist.cor))

get_dist(?)

help(get_dist)

??get_dist

dd<-daisy(hofstededf[c(2:5)])

round(as.matrix(dd))

fviz_dist(dd)

plot(hclust(dist.eucl, method="average"))
