# Supplementary material for: Situating zoonotic diseases in peacebuilding and development theories: Prioritizing zoonoses in Jordan
Source: PLoS One. 2022 Mar 17;17(3):e0265508. doi: 10.1371/journal.pone.0265508 (PMC8929606; doi:10.1371/journal.pone.0265508)
Supplement: S2 Appendix — (ZIP) [file pone.0265508.s002.zip › CFA and candidate variable data.docx]

**Inferences for Jordan from Candidate Variables used in Confirmatory Factor Analysis**

It can be seen clearly that in regard to nearly all of the variables we assessed related to drivers of health, the situation of Jordan is drastically different than those of the countries analyzed. Therefore, we will discuss implications of these findings for construction of our modified OHZDP tool. Table 13 shows the candidate variable data for severity of disease.

**Table 13**

*Candidate Variable Data for Severity of Disease*

|  | **Severity of Disease Weight** | **Malaria Incidence per 1,000** | **DALYs Lost to Communicable Diseases per 100,000** | **Infant Mortality per 1,000** | **Hospital beds per 1,000** |
| --- | --- | --- | --- | --- | --- |
| **Jordan** | TBD | NA | 3509 | 13 | 1.5 |
| **Burkina Faso** | 0.35 | 389 | 29540 | 54 | 0.4 |
| **Cameroon** | 0.2 | 264 | 26230 | 50 | 1.3 |
| **Côte d'Ivoire** | 0.2099 | 349 | 26925 | 59 | 0.4 |
| **ECOWAS** | 0.36 | 381 | 31191 | 74 | 0.5 |
| **Kenya** | 0.23 | 166 | 21359 | 32 | 1.4 |
| **Mali** | 0.35 | 449 | 32314 | 60 | 0.1 |
| **Mozambique** | 0.286 | 298 | 36015 | 55 | 0.7 |
| **Tanzania** | 0.21 | 114 | 20328 | 36 | 0.7 |
| **Uganda** | 0.21 | 218 | 22467 | 33 | 0.5 |

*Note.* Table made by author. Sources:

Malaria: <https://ourworldindata.org/malaria>

DALYS: <https://ourworldindata.org/burden-of-disease>

Infant: <https://data.worldbank.org/indicator/SP.DYN.IMRT.IN>

Hosp beds:<http://data.un.org/Data.aspx?d=WHO&f=MEASURE_CODE%3AWHS6_102>

Hosp beds: <https://www.cia.gov/library/publications/the-world-factbook/fields/360.html>

Jordan has significantly lower rates of infant mortality, as well as DALYs lost to communicable diseases. It is worth noting that infant mortality rates in most Middle East countries are generally lower than those in Africa. This could indicate severity of disease may warrant a lower weighting for Jordan than what many of the African countries assigned it. In the same vein, Jordan has higher hospital beds per 1,000 people, which according to our analysis may be a reason for a lower weighting as well. Notably, there is no malaria data for Jordan as it is considered a WHO certified malaria free country (WHO, 2019c). However, given that malaria incidence was considered of significance, it seems reasonable to infer that a zoonotic disease endemic in Jordan may be useful to consider when assigning weight to severity of disease.

Table 14 shows the candidate variable data for the socioeconomic and environmental criteria.

**Table 14**

*Candidate Variable Data for Socioeconomic and Environmental*

|  | **Socioeconomic and Environmental Weight** | **Percent of population living in rural area** | **Sugar consumption per capita** | **Democratic Index** | **Percent of GDP Comprised of Agriculture** |
| --- | --- | --- | --- | --- | --- |
| **Jordan** | TBD | 0.08 | 33.1 | 3.62 | 0.03 |
| **Burkina Faso** | 0.15 | 0.7 | 8.03 | 3.73 | 0.2 |
| **Cameroon** | 0.198 | 0.43 | 7.51 | 2.77 | 0.15 |
| **Côte d'Ivoire** | 0.1935 | 0.49 | 8.91 | 4.11 | 0.21 |
| **ECOWAS** | 0.12 | 0.49 | 8.44 | 4.10 | 0.22 |
| **Kenya** | 0.21 | 0.72 | 14.4 | 5.05 | 0.34 |
| **Mali** | 0.13 | 0.57 | 9.04 | 3.93 | 0.37 |
| **Mozambique** | 0.11 | 0.63 | 10.08 | 3.51 | 0.26 |
| **Tanzania** | 0.2 | 0.66 | 9.55 | 5.10 | 0.29 |
| **Uganda** | 0.19 | 0.76 | 11.7 | 4.94 | 0.23 |

*Note.* Table made by author. Sources:

Rural: <https://data.worldbank.org/indicator/SP.RUR.TOTL.ZS>

Sugar: <https://www.helgilibrary.com/indicators/sugar-consumption-per-capita/>

DI:<https://www.eiu.com/n/campaigns/democracy-index-2020/#mktoForm_anchor>

Agriculture: <https://data.worldbank.org/indicator/NV.AGR.TOTL.ZS?view=chart>

Jordan has a much higher sugar consumption and significantly lower population living in rural areas which speaks to its upper-middle income status. As such, this suggests that weight allotment may be higher for Jordan than the other countries analyzed. In regard to democratic index, Jordan was relatively average among the countries. Higher democratic index scores were positively correlated with weight, so this too may suggest a higher weight allotment in this criterion for Jordan. Notably, Jordan has a much lower GDP comprised of agriculture, which could suggest a lower weight allotment, however this variable was not found to be statistically significant and will therefore be considered with caution.

Candidate variable data for disease burden can be seen in Table 15.

**Table 15**

*Candidate Variable Data for Disease Burden*

|  | **Disease Burden Weight** | **Bovine Meat consumption per kg/capita/year** | **Milk consumption per capita** | **Deaths from unsafe sanitation per 100,000** | **Cattle per capita** |
| --- | --- | --- | --- | --- | --- |
| **Jordan** | TBD | 5.61 | 77.68 | 0.09 | 0.007 |
| **Burkina Faso** | 0.33 | 4.48 | 29.79 | 52 | 0.47 |
| **Cameroon** | 0.4 | 4.17 | 15.27 | 38 | 0.22 |
| **Côte d'Ivoire** | 0.4 | 1.7 | 6.36 | 34 | 0.06 |
| **ECOWAS** | 0.18 | 3.45 | 7.91 | 39 | 0.1 |
| **Kenya** | 0.39 | 9.54 | 94.86 | 48 | 0.34 |
| **Mali** | 0.26 | 9.96 | 98.68 | 71 | 0.49 |
| **Mozambique** | 0.33 | 1.04 | 4.79 | 44 | 0.05 |
| **Tanzania** | 0.398 | 6.09 | 40.29 | 31 | 0.41 |
| **Uganda** | 0.205 | 5.15 | 37.27 | 37 | 0.28 |

*Note.* Table made by author. Sources:

Bovine: <https://www.fao.org/faostat/en/#data/domains_table>

Milk:<https://ourworldindata.org/grapher/per-capita-milk-consumption?tab=table>

Unsafe sanitation: <https://ourworldindata.org/sanitation>

Cattle: <https://www.fao.org/faostat/en/#data/domains_table>

Jordan demonstrates about median bovine meat consumption at 5.61 between a range of 1.7 - 9.96, with comparably higher milk consumption to most countries. Given milk consumption and bovine meat consumption are risk factors for zoonotic spillover, this suggests consideration of higher weight allotment. However, Jordan demonstrates much lower rates of deaths attributed to unsafe sanitation and cattle per capita. This suggests that these indicators of disease burden are less significant in Jordan compared to Africa, which could suggest a lower weighting assignment to this criterion. Given this and our findings from severity of disease candidate variables, it seems Jordan’s overall health situation and zoonotic landscape in the areas of these two criteria is less than those in the African countries, which implies that our modified tool will likely operate under a different weighting scheme.

Table 16 displays the candidate variable data for control measures.

**Table 16**

*Candidate Variable Data for Control Measures*

|  | **Control Measures Weight** | **Number (x/15) of OIE surveillance measures in place for bovine tuberculosis** | **Veterinary and para-vet professionals in the country** | **Tuberculosis mortality per 100,000** | **Percent of population under 5 using insecticide nets** |
| --- | --- | --- | --- | --- | --- |
| **Jordan** | TBD | 6 | 1835 | 0.12 | NA |
| **Burkina Faso** | 0.13 | 2 | 1669 | 7.8 | 0.75 |
| **Cameroon** | 0.198 | 1 | 4458 | 31 | 0.55 |
| **Côte d'Ivoire** | 0.1937 | 2 | 756 | 22 | 0.6 |
| **ECOWAS** | 0.19 | 3 | 15,277 | 53 | 0.49 |
| **Kenya** | 0.17 | 5 | 6631 | 38 | 0.56 |
| **Mali** | 0.17 | 0 | 1585 | 7.7 | 0.79 |
| **Mozambique** | 0.142 | 6 | 6050 | 72 | 0.48 |
| **Tanzania** | 0.186 | 5 | 5026 | 40 | 0.54 |
| **Uganda** | 0.205 | 4 | 3505 | 20 | 0.62 |

*Note.* Table made by author. Sources:

Surveillance: <https://www.oie.int/wahis_2/public/wahid.php/Diseasecontrol/measures>

TB: <https://apps.who.int/gho/data/node.main.1317>

Vets: <https://www.oie.int/wahis_2/public/wahid.php/Countryinformation/Veterinarians>

Insecticide: <https://www.indexmundi.com/facts/indicators/SH.MLR.NETS.ZS/map/africa>

Here it can be seen that Jordan has the highest number of OIE surveillance measures in place for bovine tuberculosis, along with Mozambique, which may indicate higher weight allotment for this criterion. Jordan does not have many veterinarian professionals compared to most of the other countries. It also has much lower tuberculosis mortality rates. Given Jordan is a certified malaria free country (WHO, 2019c), there is no data on the percent of population under five sleeping under insecticide nets. The fact that Jordan is on the lower end of veterinary professionals would advocate for perhaps higher weight allotment. However, the lower tuberculosis rates may suggest lowering the weight allotment.  Overall, given a direct control measure, such as insecticide nets, was found significant, another applicable variable may be useful to evaluate for Jordan when considering weight for this criterion.
