# Supplementary material for: Situating zoonotic diseases in peacebuilding and development theories: Prioritizing zoonoses in Jordan
Source: PLoS One. 2022 Mar 17;17(3):e0265508. doi: 10.1371/journal.pone.0265508 (PMC8929606; doi:10.1371/journal.pone.0265508)
Supplement: S2 Appendix — (ZIP) [file pone.0265508.s002.zip › Log Reg Anlaysis Data and Methodology.docx]

**Methodology**

 Through thematic and descriptive analyses, we found six countries, Burkina Faso, Cameroon, Côte D’Ivoire, Kenya, Mali, and Tanzania, to be operating under a similar framework, suitable to pursue multiple logistic regression analysis.

We evaluated weights from the four criteria generated from our thematic analysis: severity of disease, disease burden, socioeconomic and environmental, and control measures. For statistical analysis, we used the criteria with the smallest weight, control measures, for the baseline. Each criteria weight was inflated so that the sum was  1 except for Kenya, whose weight summed 1 without inflation. Then, as the dependent variable, the log odds of the adjusted weight of the three categories, severity of disease, socioeconomic and environmental, and disease burden, were taken in relation to weight of the baseline category control measures. We investigated if the relative criteria identified in thematic analysis (severity of disease, disease burden, socioeconomic and environmental, and control measures) weights were allocated according to the magnitude of candidate explanatory variables in addition to those three category intercepts (for example, for socioeconomic and environmental criteria we selected the following drivers of health: GDP, GINI index, and median household income): We allowed for the possibility that each country has a predetermined value (which is the value of intercept) for each category by constructing intercepts and assumed that the deviation from those predetermined values could be explained by candidate explanatory variables.

We performed logistic regression analysis, with a comprehensive set of combinations of one candidate explanatory variable from the three categories other than baseline: We chose one explanatory variable from each of the three categories in addition to the three intercepts. This left us with 12C3=220 possibilities. However, we found that only the intercepts demonstrated significance, suggesting that none of the explanatory variables explain deviation from the intercept.  Therefore, we inferred the weight determination follows a more complicated mechanism and then investigated each criterion separately by means of confirmatory factor analysis.

**Candidate Variables for Logistic Regression Raw Data**

*Candidate Variables for Logistic Regression: Severity of Disease and Disease Burden*

|  | **Severity of Disease** | | | | **Disease Burden** | | | |
| --- | --- | --- | --- | --- | --- | --- | --- | --- |
|  |  |  |  |  |  | |  | |
|  | Weight | TB mortality per 100,000 in 2018 | Malaria mortality rate per 100,000 2017 | HIV mortality per 100,000 2017 | Weight | Hospital beds per person per 1000 people | Domestic general gov health exp per capita in USD | Current Health Expenditure per capita in USD |
| **Burkina Faso** | 0.35 | 7.8 | 130 | 15.23 | 0.33 | 0.4 | 19.23 | 44.4 |
| **Cameroon** | 0.2 | 31 | 91 | 104.23 | 0.4 | 1.3 | 9.03 | 67.81 |
| **Cote D'Ivoire** | 0.2099 | 22 | 75 | 88.81 | 0.4 | 0.4 | 19.85 | 69.75 |
| **ECOWAS** | 0.36 | 53 | 69 | 110.44 | 0.18 | 5 | 10.48 | 73.92 |
| **Ethiopia** | 0.23 | 22 | 3.21 | 19.01 | 0.41 | 0.3 | 6.03 | 25.26 |
| **Kenya** | 0.23 | 38 | 12.73 | 119.57 | 0.39 | 1.4 | 32.74 | 76.61 |
| **Mali** | 0.35 | 7.7 | 95.62 | 37.7 | 0.26 | 0.1 | 10.96 | 31.38 |
| **Mozambique** | 0.286 | 72 | 71.51 | 226.84 | 0.33 | 0.7 | 6.29 | 21.07 |
| **Pakistan** | 0.406 | 20 | 2.52 | 2.79 | 0.23 | 0.6 | 14.08 | 44.59 |
| **Tanzania** | 0.21 | 40 | 29 | 63.27 | 0.398 | 0.7 | 14.67 | 33.92 |
| **Uganda** | 0.21 | 20 | 55 | 74.1 | 0.205 | 0.5 | 6.03 | 38.43 |
| **US** | 0.33 | 0.18 | 0 | 1.85 | 0.48 | 3 | 5,139.27 | 10,246.14 |
| **Jordan** | TBD | 0.12 | 0 | 0.23 | TBD | 1.5 | 152.62 | 340.66 |

*Note.* Data obtained from the following sources in October 2020:

Central Intelligence Agency (CIA). (2020). *The World Factbook.* Retrieved from https://www.cia.gov/library/publications/the-world-factbook/geos/us.html

Roser, M. & Ritchie, H. (2019a). *Malaria.* Our World in Data. Retrieved https://ourworldindata.org/malaria

Roser, M. & Ritchie, H. (2019b). *HIV/AIDS.* Our World in Data. Retrieved from https://ourworldindata.org/hiv-aids

The World Bank Data. (2018). Domestic general government health expenditure per capita (current US$). Retrieved from https://data.worldbank.org/indicator/SH.XPD.GHED.PC.CD?locations=CM

United Nations (UN) Data. (2014, July 31). Hospital beds per 10,000. Retrieved from http://data.un.org/Data.aspx?d=WHO&f=MEASURE_CODE%3AWHS6_102

World Health Organization (WHO). (2019, May 22). Countries and territories certified malaria free by WHO. Retrieved from https://www.who.int/malaria/areas/elimination/malaria-free-countries/en/

World Health Organization (WHO). (2021). *Global health observatory data repository.* Retrieved from https://apps.who.int/gho/data/node.main

*Candidate Variables for Logistic Regression: Socioeconomic and Environmental and Control Measures*

|  | **Socioeconomic and Environmental** | | | | **Control Measures** | | | |
| --- | --- | --- | --- | --- | --- | --- | --- | --- |
|  |  | | |  |  | | | |
|  | Weight | GDP per capita | GINI Index (World Bank) | Median household income | Weight | Meat consumption per capita | Vet and para-vet professionals in 2015 | Of the 15 control measures outlined by OIE: how many are in place for cattle for BTB surveillance |
| **Burkina Faso** | 0.15 | 774.8 | 35.3 | 1530 | 0.13 | 14.8 | 1669 | 2 |
| **Cameroon** | 0.198 | 1497.9 | 46.6 | 2,075 | 0.198 | 12.7 | 4458 | 1 |
| **Cote D'Ivoire** | 0.1935 | 2286.2 | 41.5 | 2,346 | 0.1937 | 13.3 | 756 | 2 |
| **ECOWAS** | 0.12 | 2229.9 | 43 | 2,667 | 0.19 | 8.8 | 15,277 | 3 |
| **Ethiopia** | ***NA*** | 857.5 | 35 | 2865 | 0.19 | 8.5 | 16,906 | 3 |
| **Kenya** | 0.21 | 1816.5 | 40.8 | 1870 | 0.17 | 16.7 | 6631 | 5 |
| **Mali** | 0.13 | 890.7 | 33 | 1983 | 0.17 | 22.2 | 1585 | 0 |
| **Mozambique** | 0.11 | 491.8 | 54 | 3576 | 0.142 | 7.8 | 6050 | 6 |
| **Pakistan** | ***NA*** | 1284.7 | 33.5 | 4,060 | 0.175 | 14.7 | 13,224 | 2 |
| **Tanzania** | 0.2 | 1122.1 | 40.5 | 2154 | 0.186 | 9.6 | 5026 | 5 |
| **Uganda** | 0.19 | 776.8 | 42.8 | 1775 | 0.205 | 11 | 3505 | 4 |
| **US** | 0.156 | 65,118.40 | 41.4 | 43585 | NA | 120.2 | 156,759 | 10 |
| **Jordan** | TBD | 4330.3 | 33.7 | 4231 | TBD | 42 | 1835 | 6 |
|  |  |  |  | note refugees: 2821 |  |  |  |  |

*Note.* Data obtained from the following sources in October 2020:

Food and Agriculture Organization (FAO). (2019). *FAOSTAT Database.* Retrieved from http://www.fao.org/faostat/en/#data/CL%20/%20http://chartsbin.com/view/12730

Phelps, G. & Crabtree, S. (2013). *Worldwide, median household income about $10,000.* Gallup. Retrieved https://news.gallup.com/poll/166211/worldwide-median-household-income-000.aspx

The World Bank Data. (2019a). *GDP per capita*. Retrieved from https://data.worldbank.org/indicator/NY.GDP.PCAP.CD?end=2019&locations=BF-CM-NG-CI-ET-KE-ML-MZ-PK-UG-TZ-US&start=1960&view=chart&year_high_desc=false

The World Bank Data. (2019b). *GINI per capita.* Retrieved from https://data.worldbank.org/indicator/NY.GNP.PCAP.CD?locations=BF-CM-NG-CI-ET-KE-ML-MZ-PK-UG-TZ-US

World Organization for Animal Health. (2020a). *Veterinarians.* World Organization for Animal Health Information System. Retrieved from https://www.oie.int/wahis_2/public/wahid.php/Countryinformation/Veterinarians

World Organization for Animal Health. (2020b). *Disease control measures.* World Organization for Animal Health Information System. Retrieved from

https://www.oie.int/wahis_2/public/wahid.php/Diseasecontrol/measures

**Multiple Logistic Regression Analysis Selected Results**

A sample of the results of this analysis of 12C3=220 possibilities can be seen below.

*Logistic Regression 1*

| Candidate Explanatory Variables / Intercepts | Estimate | Standard Error | T Value | P Value |
| --- | --- | --- | --- | --- |
| Intercept 1 (SD) | -1.384e+00 | 4.364e-01 | -3.172 | 0.00804 ** |
| Intercept 2 (SE) | -1.729e+00 | 4.364e-01 | -3.962 | 0.00189 ** |
| Intercept 3 (DB) | -1.123e+00 | 4.364e-01 | -2.574 | 0.02435 * |
| Malaria Mortality | 1.269e-03 | 2.683e-03 | 0.473 | 0.64470 |
| Median Household Income | -4.987e-03 | 4.085e-02 | -0.122 | 0.90484 |
| Vets | 4.277e-05 | 4.631e-05 | 0.924 | 0.37388 |

*Logistic Regression 2*

| Candidate Explanatory Variables / Intercepts | Estimate | Standard Error | T Value | P Value |
| --- | --- | --- | --- | --- |
| Intercept 1 (SD) | -1.1354364 | 0.2029086 | -5.596 | 0.000117 *** |
| Intercept 2 (SE) | -1.4802469 | 0.2029086 | -7.295 | 9.54e-06 *** |
| Intercept 3 (DB) | -0.8747629 | 0.2029086 | -4.311 | 0.001012 ** |
| TBM | 0.0036974 | 0.0049234 | 0.751 | 0.467131 |
| GDP | -0.0002109 | 0.0001986 | -1.062 | 0.309061 |
| CHE | 0.0032161 | 0.0057353 | 0.561 | 0.585281 |

*Logistic Regression 3*

| Candidate Explanatory Variables / Intercepts | Estimate | Standard Error | T Value | P Value |
| --- | --- | --- | --- | --- |
| Intercept 1 (SD) | -1.2564018 | 0.3611375 | -3.479 | 0.004555 ** |
| Intercept 2 (SE) | -1.6012123 | 0.3611375 | -4.434 | 0.000816 *** |
| Intercept 3 (DB) | -0.9957283 | 0.3611375 | -2.757 | 0.017369 * |
| HIVM | -0.0002832 | 0.0017797 | -0.159 | 0.876239 |
| Meat | 0.0042109 | 0.0180242 | 0.234 | 0.819214 |
| OIE | 0.0189995 | 0.0389386 | 0.488 | 0.634390 |

*Logistic Regression 4*

| Candidate Explanatory Variables / Intercepts | Estimate | Standard Error | T Value | P Value |
| --- | --- | --- | --- | --- |
| Intercept 1 (SD) | -1.1308587 | 0.3206952 | -3.526 | 0.004175 ** |
| Intercept 2 (SE) | -1.4756692 | 0.3206952 | -4.601 | 0.000609 *** |
| Intercept 3 (DB) | -0.8701852 | 0.3206952 | -2.713 | 0.018835 * |
| MM | -0.0005516 | 0.0018950 | -0.291 | 0.775960 |
| Meat | 0.0017101 | 0.0164939 | 0.104 | 0.919137 |
| GHE | -0.0011958 | 0.0094602 | -0.126 | 0.901508 |

*Logistic Regression 5*

| Candidate Explanatory Variables / Intercepts | Estimate | Standard Error | T Value | P Value |
| --- | --- | --- | --- | --- |
| Intercept 1 (SD) | -1.0729402 | 0.2087459 | -5.140 | 0.000245 *** |
| Intercept 2 (SE) | -1.4177507 | 0.2087459 | -6.792 | 1.93e-05 *** |
| Intercept 3 (DB) | -0.8122667 | 0.2087459 | -3.891 | 0.002144 ** |
| HIVM | 0.0007938 | 0.0061207 | 0.130 | 0.898958 |
| GDP | -0.0001539 | 0.0002695 | -0.571 | 0.578552 |
| Hosp | 0.0905499 | 0.3035745 | 0.298 | 0.770592 |

*Logistic Regression 6*

| Candidate Explanatory Variables / Intercepts | Estimate | Standard Error | T Value | P Value |
| --- | --- | --- | --- | --- |
| Intercept 1 (SD) | -1.2796866 | 0.4155832 | -3.079 | 0.00955 ** |
| Intercept 2 (SE) | -1.6244971 | 0.4155832 | -3.909 | 0.00208 ** |
| Intercept 3 (DB) | -1.0190131 | 0.4155832 | -2.452 | 0.03048 * |
| TBM | -0.0001652 | 0.0088153 | -0.019 | 0.98535 |
| Meat | 0.0035972 | 0.0200366 | 0.180 | 0.86052 |
| Hosp | 0.0889059 | 0.2023888 | 0.439 | 0.66826 |

All 220 results can be seen using associated R code.
