# Supplementary material for: Situating zoonotic diseases in peacebuilding and development theories: Prioritizing zoonoses in Jordan
Source: PLoS One. 2022 Mar 17;17(3):e0265508. doi: 10.1371/journal.pone.0265508 (PMC8929606; doi:10.1371/journal.pone.0265508)
Supplement: S3 Appendix — (DOCX) [file pone.0265508.s003.docx]

**Phase 3 Prioritization of Zoonoses in Jordan with Modified Tool: Raw Data**

 
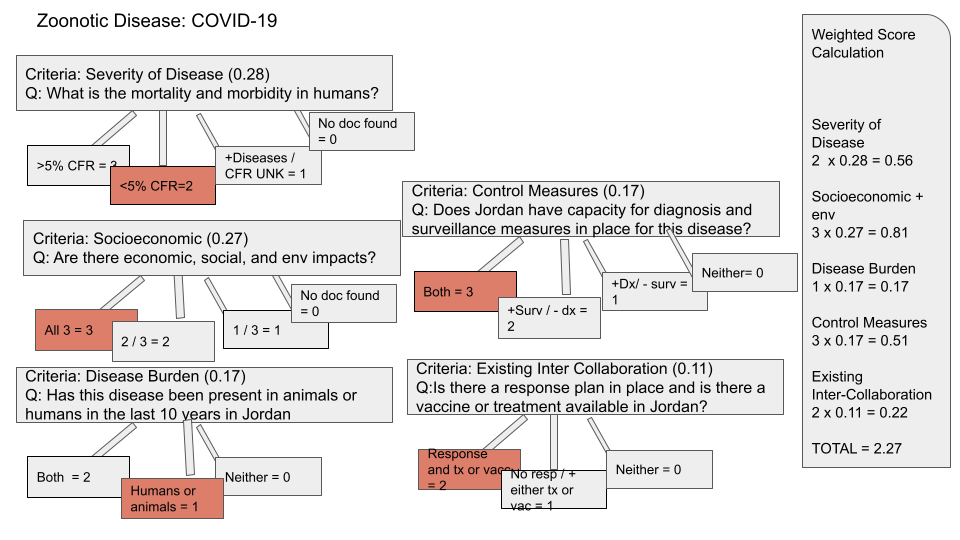


SD:  CFR = deaths / confirmed cases. Feb: 4,395/338,322 in Jordan = 0.013. Global =0.023 -> less than 5%. WHO COVID Dashboard (<https://covid19.who.int/>)

SE = Social impacts: lockdowns, with surveys suggesting quality of life impacted. Environmental impacts: increase in plastics, sanitizing chemicals, increase in medical waste. Economic impacts: unemployment, drop in GDP, disrupt tourism, disproportionately impacting refugees.

<https://reliefweb.int/report/jordan/jordan-and-covid-19-effective-response-high-cost>

<https://wrmcouncil.org/wp-content/uploads/2020/11/Jordan_Economic-Impact_Istaiteyeh_WRMC_Nov2020.pdf>

<https://www.jo.undp.org/content/jordan/en/home/presscenter/articles/2020/covid-19-and-the-environment-impact-and-response--.html>

DB= Documented human cases (<https://covid19.who.int/>); no animals in Jordan thus far per OIE database.

***This is the OIE database citation for all the following OIE database references:**

World Organization for Animal Health (OIE). (n.d.). *World Animal Health Information*

*Database (WAHIS Interface).* Retrieved October 15, 2020 from [https://www.oie.int/wahis_2/public/wahid.php/Countryinformation/Zoonose](https://www.oie.int/wahis_2/public/wahid.php/Countryinformation/Zoonoses)

CM= Ability to diagnose, and other control measures including surveillance, quarantine, etc… in place.

<https://reliefweb.int/report/jordan/jordan-and-covid-19-effective-response-high-cost>

EC= A vaccine is in place, as well as a response plan.

https://www.unhcr.org/news/press/2021/1/5ffffe614/refugees-receive-covid-19-vaccinations-jordan.html

 
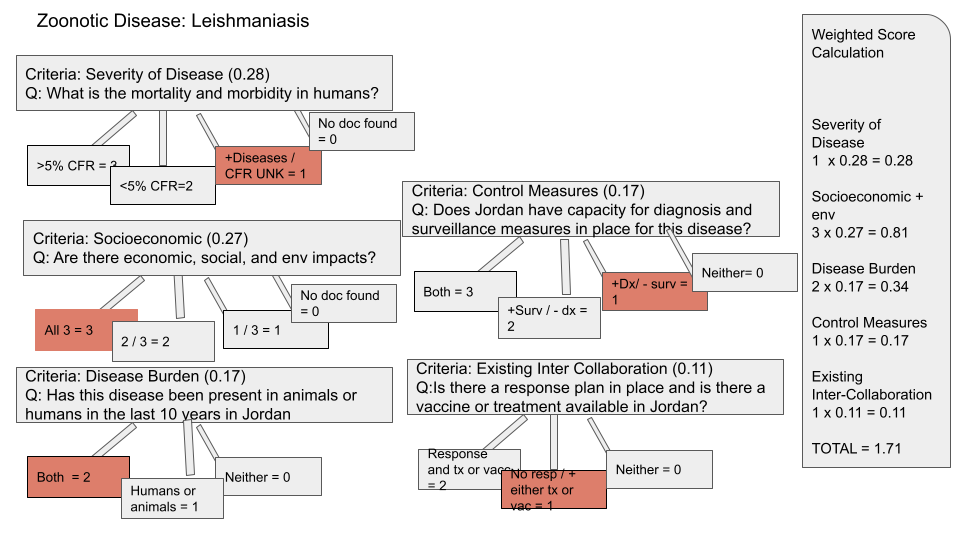


SD:  CFR not found for cutaneous leishmaniasis, which is the most prevalent form worldwide and in Jordan.

<https://link.springer.com/article/10.1007%2Fs12061-014-9113-3>

SE = Social impacts include stigma, scarring with quality-of-life impacts. Environmental impacts include sandflies (vector control) and migration. Economic impacts are that most people impacted are refugees and care offered is often provided at no cost to the individual.

<http://www.jordantimes.com/news/local/200-cases-leishmaniasis-recorded-jordan-annually> / <https://www.ncbi.nlm.nih.gov/pmc/articles/PMC7139431/>

DB= Seen in both human and animal populations per OIE database.

CM= Diagnostics available, no surveillance in place.

<https://reliefweb.int/sites/reliefweb.int/files/resources/Technical_Report_Leishmaniasis_Jordan.pdf>

EC= No response plan found. There is a treatment available from MoH in Jordan provided to the camps upon request. <https://reliefweb.int/sites/reliefweb.int/files/resources/Technical_Report_Leishmaniasis_Jordan.pdf>


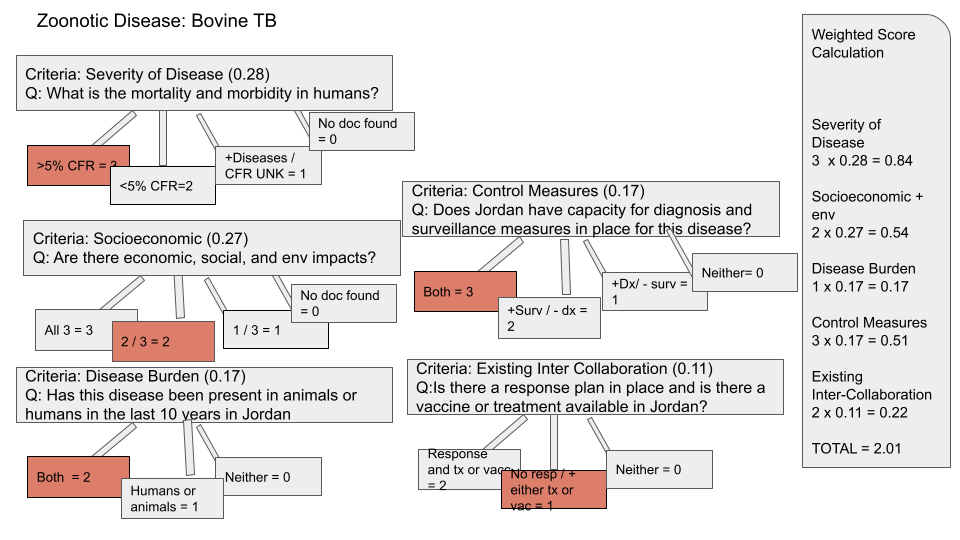


SD: 5.2% CFR.

<https://wwwnc.cdc.gov/eid/article/17/3/10-1111_article>

<https://www.ncbi.nlm.nih.gov/pmc/articles/PMC3549981/>

SE = Lack of data regarding Jordan specifically, however, globally: economic impacts including loss of livestock, environmental impact is that animal populations found with this disease undergo culling in many countries.

<https://tbfacts.org/bovine-tb/>

DB= Seen in humans and animals per OIE database.

CM= Active surveillance is in place, and there is capacity for diagnosis per OIE database.

EC= No resp plan found, however treatment is available.

<https://tbfacts.org/bovine-tb/>


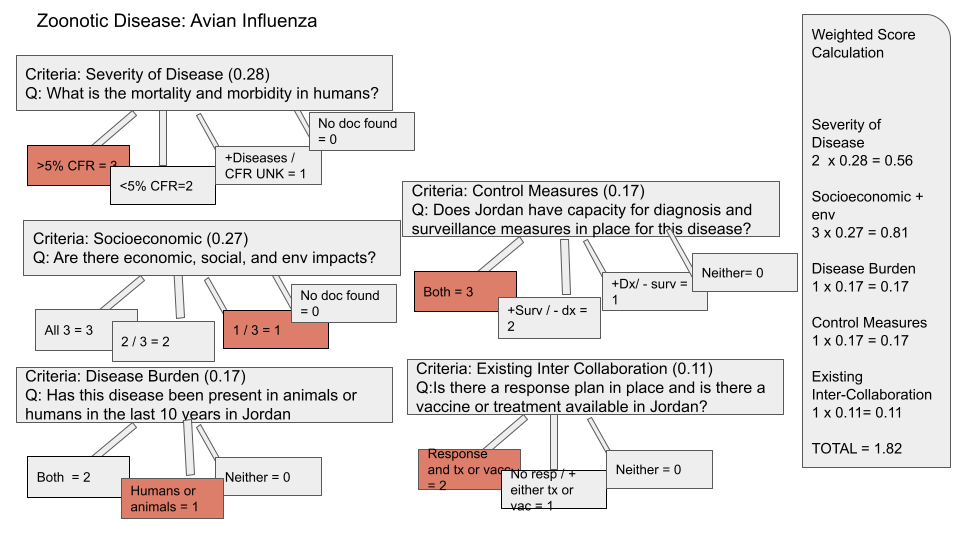


SD: The overall case-fatality rate was 56%

[https://www.who.int/csr/don/2006_06_30/en/](%20https://www.who.int/csr/don/2006_06_30/en/)

SE = 2006 outbreak resulted in economic loss in Jordan ($US169 million). No social or environmental impacts found. <https://www.researchgate.net/publication/233980165_H5N1_influenza_outbreak_during_March_2006_in_Jordan>

DB= Animals yes. No human cases in last 10 years per OIE database and

<https://www-sciencedirect-com.othmer1.icu.ac.jp:2443/science/article/pii/S003257911932512X?via%3Dihub>

CM= Surveillance and diagnostics available

<https://www.pbs.org/wgbh/nova/article/mers-jordan/>

EC= Response plan found here  [https://moh.gov.jo/EchoBusV3.0/SystemAssets/communicable/Final%20Jordan%20%20national%20preparedness%20plan%2016_10_2017.pdf](%20https://moh.gov.jo/EchoBusV3.0/SystemAssets/communicable/Final%20Jordan%20%20national%20preparedness%20plan%2016_10_2017.pdf) /

Antiviral treatment is available

<https://www.mayoclinic.org/diseases-conditions/bird-flu/diagnosis-treatment/drc-20368456>


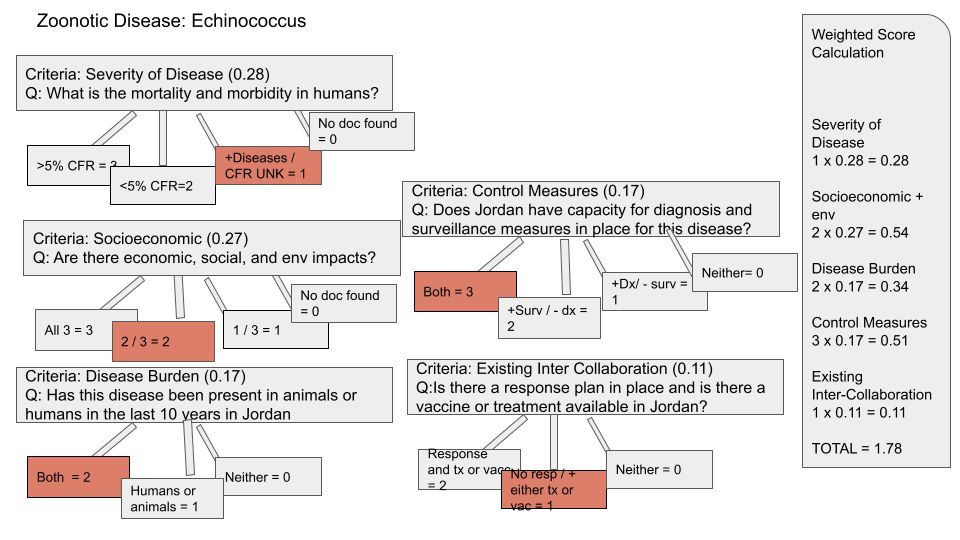


SD:  CFR – no one number found. However, in case studies conducted in Armenia and Italy, the CFR very low, but not enough data to state definitively.

<https://www.ncbi.nlm.nih.gov/pmc/articles/PMC5678460/>  [https://journals.plos.org/plosntds/article?id=10.1371/journal.pntd.0005771](%20https://journals.plos.org/plosntds/article?id=10.1371/journal.pntd.0005771)

SE = Demonstrated economic losses, and livestock production losses; social impacts include significant disabilities from the diagnosis

<https://link.springer.com/article/10.1007/s00436-003-0883-9>

<https://www.researchgate.net/publication/7282122_Global_Socioeconomic_Impact_of_Cystic_Echinococcosis>

<https://www.researchgate.net/publication/352088885_Incrimination_of_Dog_Vector_of_Cystic_Echinococcosis_and_Impact_of_the_Appropriate_Dogs'_Treatment>

DB= Seen in both humans and animals per OIE database.

CM= Diagnostic capacity and surveillance in place (per OIE database)

<https://www.researchgate.net/publication/321731902_Cystic_echinococcosis_in_Jordan_A_review_of_causative_species_previous_studies_serological_and_radiological_diagnosis>

<https://www.ijmrhs.com/medical-research/a-10year-retrospective-study-on-hydatid-disease-in-jordan-with-emphasis-on-the-role-of-imaging-in-its-diagnosis.pdf>

EC= No response plan found. Treatment available [https://www.cdc.gov/parasites/echinococcosis/treatment.html](%20https://www.cdc.gov/parasites/echinococcosis/treatment.html)


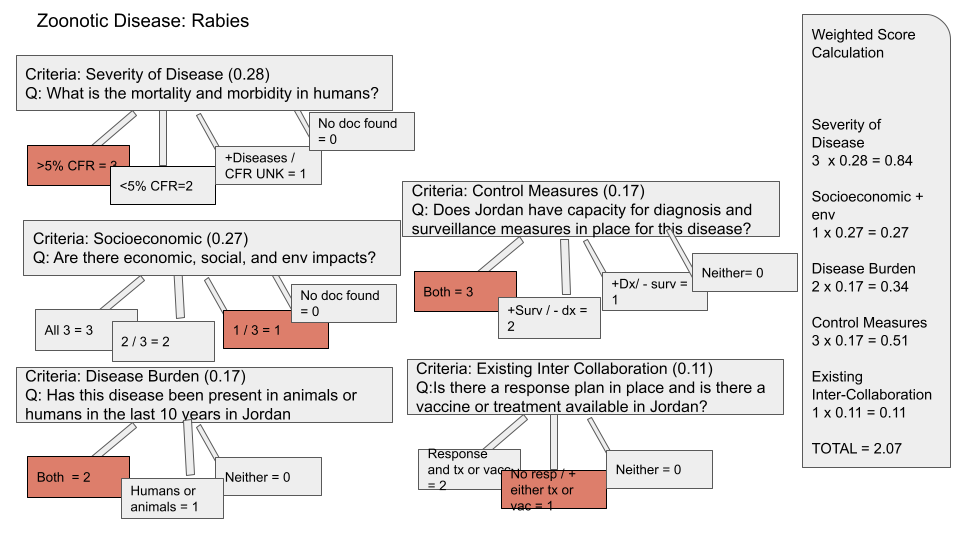


SD:  CFR 100% when untreated; most cases are not treated

<https://link.springer.com/chapter/10.1007%2F978-3-030-21084-7_1>

SE = Significant economic or environmental impacts in Jordan not found; however, social impacts given the religious element to managing rabies and dogs in Jordan

[https://www.theatlantic.com/international/archive/2017/11/jordans-holy-war-on-dogs/546401/](%20https://www.theatlantic.com/international/archive/2017/11/jordans-holy-war-on-dogs/546401/)

DB= Seen in humans and animals in Jordan per OIE database

CM= Surveillance in place; diagnostic capacity in place [https://www.oie.int/fileadmin/Home/eng/Publications_%26_Documentation/docs/pdf/TT/2015_MO1_Aidaros.pdf](%20https://www.oie.int/fileadmin/Home/eng/Publications_%26_Documentation/docs/pdf/TT/2015_MO1_Aidaros.pdf)

EC= No response plan found. Treatment is available.

<https://www.oie.int/fileadmin/Home/eng/Publications_%26_Documentation/docs/pdf/TT/2015_MO1_Aidaros.pdf>


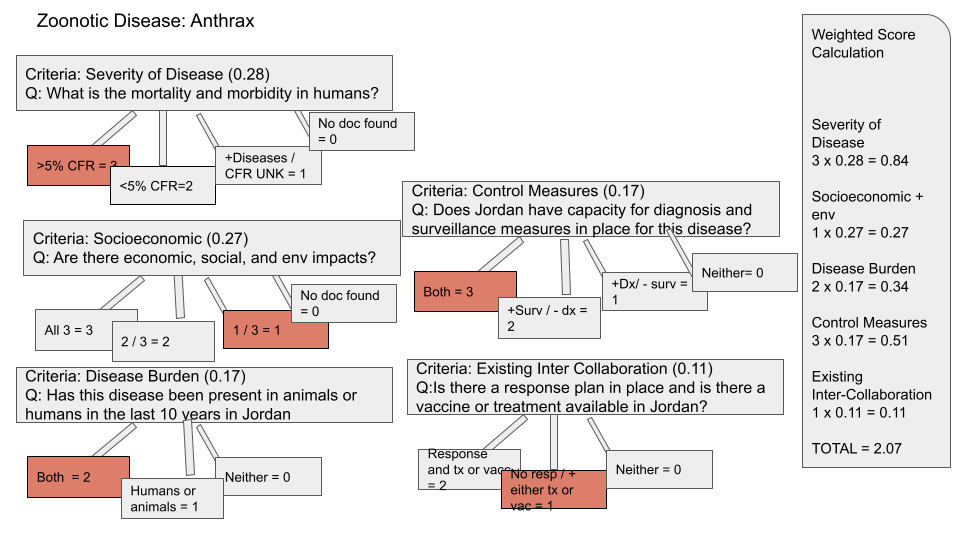


SD: 20% for cutaneous anthrax without antibiotics and 25 - 75% for gastrointestinal anthrax; inhalation anthrax has a fatality rate that is 80% or higher.

<https://www.cdc.gov/mmwr/preview/mmwrhtml/rr4915a1.htm>

<https://www.fda.gov/vaccines-blood-biologics/vaccines/anthrax>

SE = Sporadic, few cases in Jordan; no significant economic or social impacts found. Environmental impact: animals are routinely vaccinated; sometimes culling

<https://www.researchgate.net/profile/Amin-Aqel-2/publication/279990486_Molecular_characterization_of_the_circulating_Bacillus_anthracis_in_Jordan/links/55b8a63108aed621de05f47b/Molecular-characterization-of-the-circulating-Bacillus-anthracis-in-Jordan.pdf>

DB= Seen in humans and animals in Jordan per OIE database.

CM=  Surveillance in place per OIE database, diagnostic capacity in place <https://books.google.co.jp/books?id=kvvVDwAAQBAJ&pg=PA18&lpg=PA18&dq=jordan+anthrax&source=bl&ots=JQ27DgoNFf&sig=ACfU3U1YIFBY5fRN0HCgLiVHGkpaMnrhqg&hl=en&sa=X&ved=2ahUKEwitrd2qt8DtAhXFdt4KHQiTB9MQ6AEwBHoECAIQAg#v=onepage&q=jordan%20anthrax&f=false>

EC= No response plan found. Treatment with antibiotics available. Vaccine for animals.

<https://www.sciencedirect.com/science/article/abs/pii/S1090023313004140>


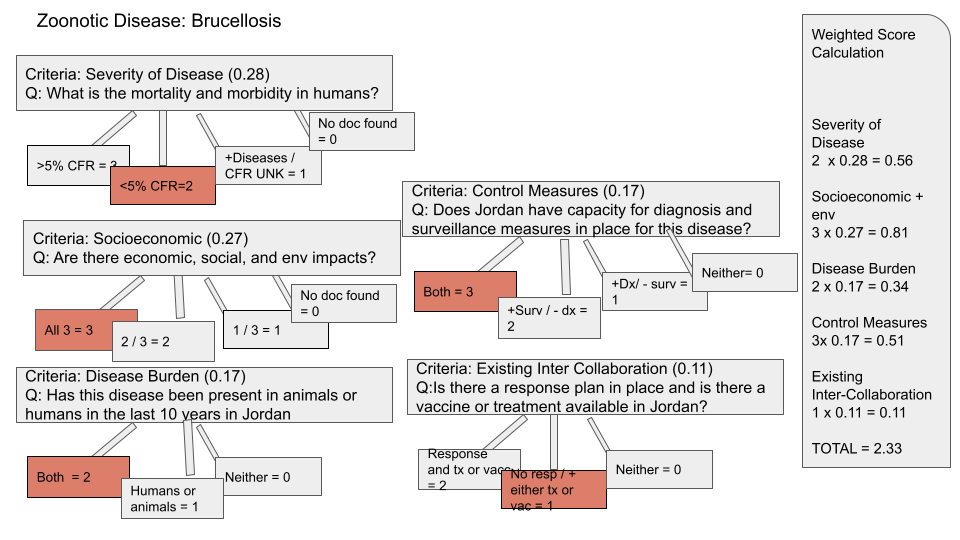


SD:  Less than 2%

[https://www.gov.mb.ca/health/publichealth/cdc/protocol/brucellosis.pdf](%20https://www.gov.mb.ca/health/publichealth/cdc/protocol/brucellosis.pdf)

SE = Economic losses from livestock deaths, social through promotion of poverty. Environmental impact, easily spreads to other animals and infected discharge from animals can harm environment it comes into contact with.

<http://pubs.sciepub.com/ajeid/5/2/2/.> [https://journals.plos.org/plosntds/article?id=10.1371/journal.pntd.0008071](%20https://journals.plos.org/plosntds/article?id=10.1371/journal.pntd.0008071)

DB= Present in both humans and animals per OIE database.

CM= Diagnostic capacity and surveillance in place

<https://apps.who.int/iris/bitstream/handle/10665/254508/WHO-WHE-CPI-2017.01-eng.pdf;sequence=1>

EC= Treatment with antibiotics is available. A vaccine is only available for animals, not humans.

<http://pubs.sciepub.com/ajeid/5/2/2/>


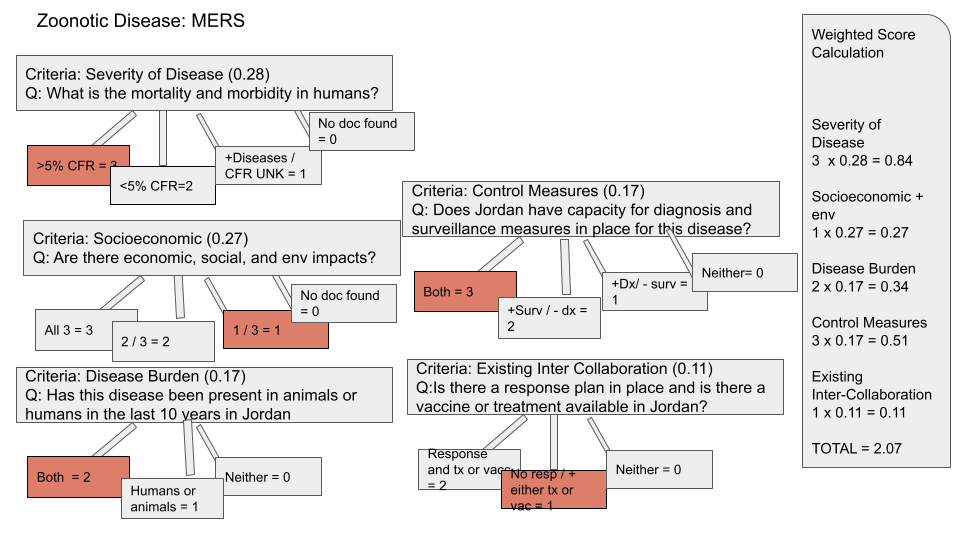


SD:  Crude CFR 34.8%

<https://www.who.int/emergencies/mers-cov/risk-assessment-july-2017.pdf?ua=1>

SE = Could not find documented evidence of significant economic impact or inferred impact (such as culling of camels etc…) or environmental impacts. However, there is literature regarding the stigma people who contracted MERS faced, suggesting a social impact.

<https://www.sciencedirect.com/science/article/pii/S1876034117301545>

DB= Present in humans: [https://www.ncbi.nlm.nih.gov/pmc/articles/PMC4806954/?tool=pmcentrez&report=abstract](%20https://www.ncbi.nlm.nih.gov/pmc/articles/PMC4806954/?tool=pmcentrez&report=abstract)

And animals<https://www.pbs.org/wgbh/nova/article/mers-jordan/>

CM=  PCR tests available for diagnosing and surveillance measures are in place.  [https://www.pbs.org/wgbh/nova/article/mers-jordan/](%20https://www.pbs.org/wgbh/nova/article/mers-jordan/)

EC= There is no current treatment (only supportive management) or vaccine available.  [https://www.nejm.org/doi/full/10.1056/NEJMsr1408795](%20https://www.nejm.org/doi/full/10.1056/NEJMsr1408795) / Jordan does have a response plan in place <https://www.pbs.org/wgbh/nova/article/mers-jordan/>


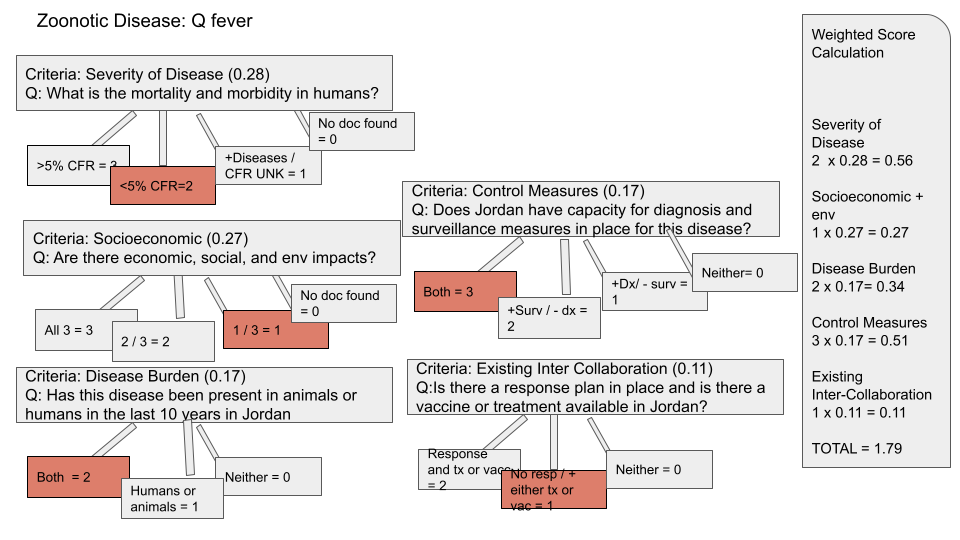


SD:  CFR less than 2%

<https://www.cdc.gov/mmwr/preview/mmwrhtml/rr6203a1.htm>

<https://www.thelancet.com/journals/laninf/article/PIIS1473-3099(05)70052-9/references>

SE = Inferred economic impact, particularly to livestock keepers, as Q fever is known to cause abortions in animals. Did not find evidence of social or environmental impacts in Jordan documented.

<http://www.pvj.com.pk/pdf-files/18_1/43-45.pdf>

DB= Present in both humans and animals in Jordan per OIE database and

<https://www.ncbi.nlm.nih.gov/pmc/articles/PMC6609193/?tool=pmcentrez&report=abstract>

CM= Active surveillance in place per OIE database. In addition, diagnostic capacity is available.

OIE database and <https://www.ajtmh.org/content/journals/10.4269/ajtmh.19-0049?crawler=true>

<https://www.ncbi.nlm.nih.gov/pmc/articles/PMC6609193/>

EC= No response plan found, however there is treatment available with antibiotics

<https://www.cdc.gov/qfever/treatment/index.html>

<https://jamanetwork.com/journals/jamanetworkopen/fullarticle/2698081>


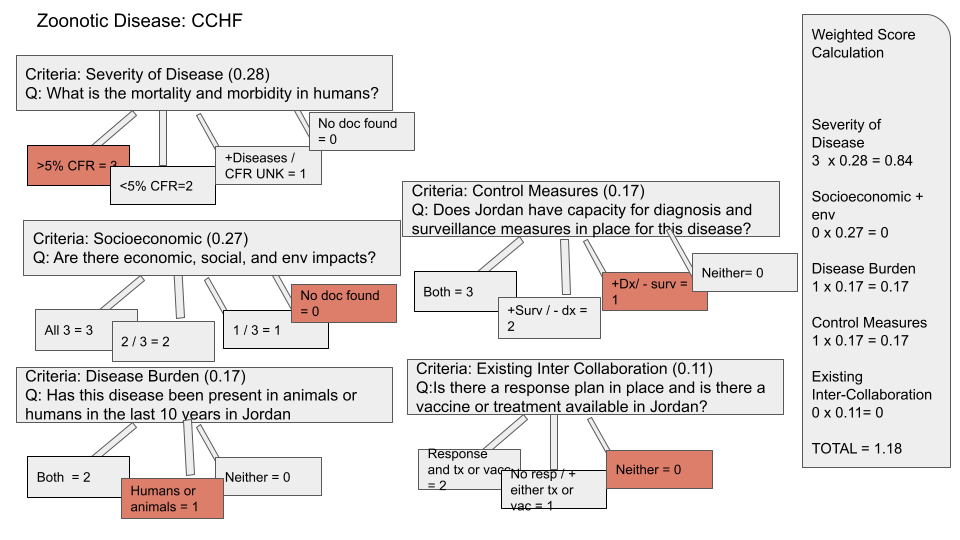


SD:  CFR is high at 40%.

<https://www.who.int/news-room/fact-sheets/detail/crimean-congo-haemorrhagic-fever>

SE = None at this point, as CCHF is a relatively new finding in animals in Jordan.

<https://www.ncbi.nlm.nih.gov/pmc/articles/PMC6335890/>

DB= Present in animals; no documented human cases in Jordan yet.

[https://www.ncbi.nlm.nih.gov/pmc/articles/PMC6335890/](%20https://www.ncbi.nlm.nih.gov/pmc/articles/PMC6335890/)

CM=Serological and antibody testing available, no active surveillance in place. <https://www.sciencedirect.com/science/article/pii/S1201971217300656>

EC= No treatments (other than supportive care) or vaccines available.

[https://www.sciencedirect.com/science/article/pii/S1201971217300656](%20https://www.sciencedirect.com/science/article/pii/S1201971217300656)
